# Impact of the use and efficacy of long lasting insecticidal net on malaria infection during the first trimester of pregnancy - a pre-conceptional cohort study in southern Benin

**DOI:** 10.1186/s12889-018-5595-2

**Published:** 2018-06-01

**Authors:** Cornélia Hounkonnou, Armel Djènontin, Seun Egbinola, Parfait Houngbegnon, Aziz Bouraima, Christophe Soares, Nadine Fievet, Manfred Accrombessi, Emmanuel Yovo, Valérie Briand, Gilles Cottrell

**Affiliations:** 1 UMR216-MERIT, French National Research Institute for Sustainable Development (IRD), Université Paris Descartes, 75006 Paris, France; 2 Centre d’Etude et de Recherche sur le Paludisme Associé à la Grossesse et à l’Enfance (CERPAGE), Faculté des Sciences de la Santé, Cotonou, Bénin; 3 Faculté des Sciences et Techniques, Université d’Abomey-Calavi, Cotonou, Bénin; 4 Centre de Recherche Entomologique de Cotonou, Cotonou, Benin; 5 University of Ibadan, Ibadan, Nigeria

**Keywords:** Gestational malaria, First trimester, Long lasting impregnated nets, Use, Physical integrity indicator, Biological efficacy indicator

## Abstract

**Background:**

Malaria in pregnancy is prevalent in Sub-Saharan Africa. The first trimester of pregnancy is a critical period and the best preventive measure is Long Lasting Insecticidal Nets (LLIN). Unfortunately, few studies have been conducted which focuses on the usage and efficacy of LLIN on malaria prevention during the first trimester.

**Methods:**

We assessed the use and effectiveness of LLIN in early pregnancy in Benin and its impact on malaria infection risk. We followed-up a cohort of 240 pregnant women from pre-conception to the end of the first trimester of pregnancy in Southern Benin. Parasitological, maternal and LLIN data were actively collected before, at the beginning and end of the first trimester of pregnancy. A Cox regression model was used to determine the relationship between the time to onset of the first malaria infection and the use, physical integrity, and bio-efficacy of the LLIN, adjusted for relevant covariables.

**Results:**

The good use, good physical integrity and biological efficacy of LLIN were associated with a decreased risk of occurrence of the first malaria infection in early pregnancy (HRa = 0.38; (0.18–0.80); *p* < 0.001; HRa = 0.59; (0.29–1.19); *p* < 0.07; HRa = 0.97; (0.94–1.00); *p* < 0.04 respectively), after adjustment for other covariates. Primi/secundigravidity and malaria infection before pregnancy were associated with a risk of earlier onset of malaria infection.

**Conclusion:**

The classically used LLIN’s indicators of possession and use may not be sufficient to characterize the true protection of pregnant women in the first trimester of pregnancy. Indicators of physical integrity and bio-efficacy should be integrated with those indicators in evaluation studies.

**Electronic supplementary material:**

The online version of this article (10.1186/s12889-018-5595-2) contains supplementary material, which is available to authorized users.

## Background

Malaria in pregnancy results in an increased risk of low birth weight (LBW) [1–3], maternal anemia and perinatal mortality [1, 4]. World Health Organization (WHO) has implemented a prevention policy specific to this vulnerable population [5, 6], based on sulfadoxine-pyrimethamine (SP) intermittent preventive treatment in pregnant women (IPTp) and the use of Long Lasting Impregnated Nets (LLIN) from the onset of pregnancy. The IPTp comprises monthly intakes of a curative dose of SP during antenatal care visits from the second trimester of pregnancy for all pregnant women (whether infected or not). Numerous studies have shown the effectiveness of IPTp in improving maternal and child health outcomes [7–10]. However, IPTp is contraindicated in the first trimester of pregnancy leaving women in the first trimester unprotected by this control tool. Moreover, several studies have shown that the first trimester of pregnancy is a critical period during which malaria infection is associated with an increased risk of low birth weight and maternal anemia [11–13]. LLIN is the only preventive tool available for pregnant women during this period. However, it is known that pregnant women attend the maternity clinic mostly after the first trimester of pregnancy in sub-Saharan Africa [14]. Consequently, the first trimester of pregnancy is a period badly covered by the malaria prevention policy. There is evidence for the efficacy of LLIN in preventing malaria infection during pregnancy [15], to improve babies’ birth weight [16]. No study has yet focused on the specific protection conferred by LLIN in the first trimester of pregnancy. In this work, using data from the follow-up of a pre-conceptional cohort carried out in South-Benin in 2015–1016, we evaluated the association between the indicators of use, physical integrity and bio-efficacy of LLINs used by pregnant women and malaria incidence during the first trimester of pregnancy.

## Methods

### Study design

EVALMOUS is a cohort study nested in the RECIPAL project, carried out in South Benin [17]. Between June 2014 and August 2017, 1214 women of childbearing age and willing to become pregnant (primary cohort) were included in a cohort follow-up. All women were screened for malaria using thick blood smear (TBS) at their inclusion and monitored monthly using a urinary pregnancy test until the identification of 411 pregnant women among them (secondary cohort). The pregnant women were then monitored monthly until delivery at the maternity clinic, where they benefited from a clinical, parasitological (by TBS), nutritional and ultrasound follow-up. Lambarene technique was used to quantify parasitaemia and the detection threshold with this method has been estimated to be 5 parasites/μL [18]. In addition, in the event of fever or symptoms suggestive to malaria, pregnant women were screened using Rapid Diagnostic Tests (RDT) and treated with Artemisinin-based Combination Therapies (ACT) when tested positive for malaria according to the national guidelines [19].

EVALMOUS study held between 1st June 2015 and 31st October 2016 and aimed to assess the effectiveness of mosquito nets used by pregnant women and other members of their household in preventing malaria. The first 576 women from the RECIPAL primary cohort who agreed to participate were included in EVALMOUS study before their pregnancy and the first 240 of them to become pregnant were followed throughout the first trimester of pregnancy. Women were visited at three home visits: a first visit was performed before pregnancy and two visits during the first trimester of pregnancy. During these visits, a questionnaire was administered to the women in order to evaluate the indicators related to LLIN, namely: possession, use and physical integrity. At the last visit the mosquito net used by the pregnant women was taken for laboratory testing to assess their bio-efficacy and replaced by new ones provided by the study. Physical integrity and bio-efficacy of the LLINs were determined by the field workers using the standard WHO protocol [20].

### Variables

#### Outcome variable

Malaria infection during the first trimester of pregnancy was defined as a positive blood smear and/or a positive RDT before 15 weeks of gestation (estimated by early ultrasound scan). Timing of malaria infection was determined based on gestational age.

#### Independent variables

At each visit during the study (before pregnancy and during the first trimester of pregnancy), the use of mosquito net was defined as a binary variable, “good” if LLIN was reported to be used every day of the week preceding the visit and if it was properly installed after inspection by the investigator.

The physical integrity of nets was characterized by a hole index (hi) resulting from the characterization of the holes according to WHO protocol [20]. A mosquito net with a hi between 0 and 64 was considered as in “good” condition, a hi between 65 and 642 as in an “acceptable” state and a hi greater than 643 as in a “bad” state.

Bio-efficacy of LLIN was based on both kd60 (knock-down 60 min) and 24 h-death (mortality after 24 h) of female *Anopheles gambiae* “Kisumu strain” 2–5 days after exposure to LLIN through standard WHO cone. A LLIN was declared bio-effective when the kd60 rate induced by this LLIN is greater than or equal to 95% or when the mortality rate induced by it is greater than or equal to 80% [20].

### Statistical analysis

First, we did a descriptive analysis of the general characteristic of the women at baseline (sociodemographic, the characteristics of the LLINs and the malaria before pregnancy).

#### Univariable analysis

We studied the relationship between the time to onset of the first malaria infection in the first trimester of pregnancy and each of the independent variables by a univariable Cox regression model. For each covariate, the proportional risk hypothesis was verified using the overall “p” of the Schoenfeld residue-based test. For the continuous covariates, the linearity hypothesis was verified. The comparison between the Kaplan-Meier curves of the different categories of qualitative variables was carried out by a log rank test.

#### Multivariable analysis

The malaria infection and the LLINs indicators were available for 190 pregnant women out of the 240. We compared the 190 pregnant women included in the multivariable analysis and the 50 excluded women according to the age (t-test), the gestational rank, the residence area and the malaria infection before pregnancy. The comparison of the age in the two groups was performed by a t-test whereas the categorical variables were compared using a Chi2 test.

A multivariable Cox regression model was used. Adjustment factors were age (in years) of the pregnant women (quantitative variable recoded in quartiles); the gestational rank (less than 3 pregnancies/more than 3 pregnancies; marital status (unmarried/married); pre-pregnancy malaria infection during the pre-pregnancy visit; ethnicity (Toffin/other); level of education (literate/illiterate); occupational status (employed/unemployed) and residential area (lake area/land area).

First, all covariates were introduced in the model and a backward step-by-step strategy was performed to eliminate non-significant cofactors. At the end of the analysis, only the cofactors associated with the variable to be explained at the 5% *p*-value threshold were retained in the final model in addition to the LLIN indicator variables. As we considered in this study one-sided alternative hypotheses (i.e. better use, better physical integrity and better bio-efficacy would confer protection against malaria), we used one-sided *p*-values with a threshold of significance at 5%.

During the study, six pregnant women had at least 2 malaria infections during the first trimester of pregnancy. In order to assess the determinants of the number of infections, we performed a multivariable Poisson regression model, regressing the same covariates (as in the Cox model) on the number of infections in the first trimester. In addition, in order to take into account the different number of measures between the women, an offset (the log of the number of visits) has been introduced in the model.

The analyses were carried out with STATA software version 13.1 (STATA Corporation, Texas).

## Results

**Fig. 1:**
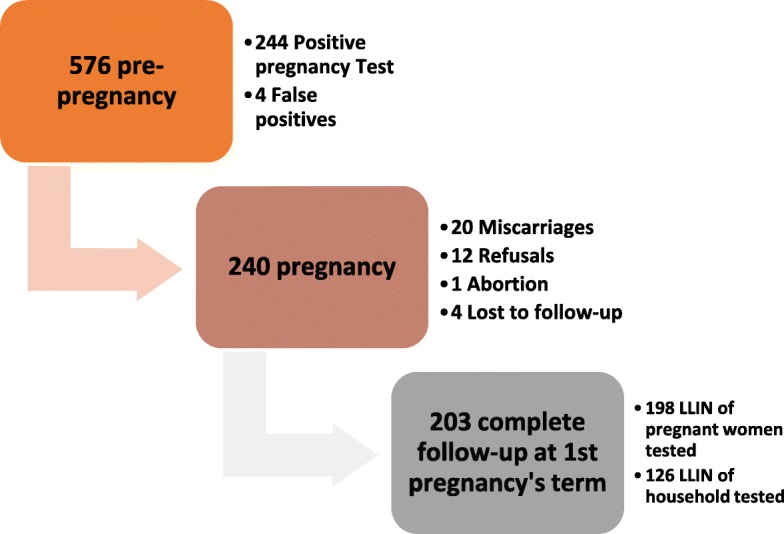
Flow chart of EVALMOUS study, Benin 2015–2016

Five hundred and seventy-six pregnant women were included in the EVALMOUS study. Figure 1 shows the flow chart during follow-up of these women. We included 240 pregnant women. At the end of the eighteen months of follow-up, the proportion of drop-out, miscarriages, and refusals was 0.42%; 8.33 and 5% respectively. The bioassays were carried out in the laboratory on 324 LLINs at the end of the first trimester.

The characteristics of the pregnant women included in the study are shown in Table 1. Among the 240 women, the prevalence of malaria infection before pregnancy was 4.41% and the proportion of infected pregnant women during the first trimester of pregnancy was 18.33%. All women except one had an LLIN.

**Table 1:** Characteristics of pregnant women at the inclusion, Sô-Ava and Akassato, Benin 2015–2016 (*N* = 240)

Characteristics	Pre-pregnancy	
	Total	Mean or proportion
		(95% CI)
Age (years)	240	26.61 ± 4.78
Gestational rank	240	
*Primigravida*	22	9.17 (5.49; 12.84)
*Secundigravida*	36	15.00 (10.45; 19.55)
*Multigravida*	182	75.83 (70.38; 81.29)
Ethnic Group	240	
*Toffin*	156	65.00 (58.92; 71.08)
*Others*	84	35.00 (28.92; 41.08)
Education level	240	
*Illiterate*	161	67.08 (61.09; 73.07)
*Literate*	79	32.92 (26.93; 38.90)
Occupational status	240	
*Employed*	221	92.08 (88.64; 95.52)
*Unemployed*	19	7.92 (4.48; 11.36)
Marital status	240	
*Cohabitation*	11	4.58 (1.92; 7.25)
*Married (monogamy)*	159	66.25 (60.22; 72.27)
*Married (polygamy)*	70	29.17 (23.37; 34.96)
Residence area	240	
*Lake area*	156	65.00 (58.92; 71.08)
*Land area*	84	35.00 (28.92; 41.08)
Pre-pregnancy malaria	227	
*Yes*	10	4.41 (1.72; 7.10)
*No*	217	95.59 (92.90; 98.28)

During pregnancy, all but one of the women had LLINs. Most of pregnant women (83.25%) had used their LLIN properly during the week prior to the visits. About 60% of the LLINs inspected were in good physical condition. On the other hand barely 6% of LLINs tested in the laboratory were bio-effective. Table 2 recapitulates the characteristics of the LLIN’s indicators inspected.

**Table 2:** Indicators of possession, use, physical integrity and chemical efficacy of LLINs inspected during follow-up, Sô-Ava and Akassato, Benin 2015–2016 (N = 240)

Characteristics	Pre-pregnancy		Pregnancy visit 1		Pregnancy visit 2	
	Total	Mean or proportion (95% CI)	Total	Mean or proportion (95% CI)	Total	Mean or proportion (95% CI)
Possession of at least one LLIN/household	240		240		203	
Yes	237	98.75 (96.17; 97.60)	239	99.53 (97.06; 99.94)	202	99.51 (96.53; 99.93)
No	3	1.25 (0.40; 3.83)	1	0.42 (0.06; 2.94)	1	0.49 (0.07; 3.47)
Possession of LLIN according to WHO standards (1 LLIN/2 people)	237		239		202	
Yes	178	75.11 (69.16; 80.23)	185	77.41 (71.63; 82.30)	164	81.19 (75.14; 86.03)
No	59	24.89 (19.77; 30.84)	54	22.59 (17.70; 28.37)	38	18.81 (13.96; 24.85)
Use of LLIN by pregnant women	237		239		202	
Yes	199	83.97 (78.69; 89.13)	203	84.94 (79.78; 88.96)	178	88.12 (82.83; 91.93)
No	38	16.03 (11.86; 21.31)	36	15.06 (11.04; 20.22)	24	11.88 (8.06; 17.17)
Physical integrity of pregnant woman’s LLIN	237		239		202	
Good	156	65.82 (59.51; 71.62)	145	60.67 (54.29; 66.70)	115	56.93 (49.95; 63.64)
Bad	81	34.18 (28.38; 40.49)	94	39.33 (33.30; 45.71)	87	43.07 (36.36; 50.05)
Physical integrity of household’s LLIN	169		195		164	
Good	101	59.76 (52.12; 66.96)	117	60.00 (52.91; 66.70)	96	58.54 (50.77; 77.90)
Bad	68	40.24 (33.04; 47.88)	78	40.00 (33.30; 47.09)	68	41.46 (34.10; 49.23)
Bio-efficacy of pregnant women’s LLIN					198	
Yes	–		–		12	6.06 (3.46; 10.41)
No	–		–		186	
Bio-efficacy of household’s LLIN					126	
Yes	–		–		7	5.56 (2.64; 11.30)
No	–		–		119	94.44 (88.70; 97.36)

Table 3 shows the different proportions of malaria infection during the different visits of the first trimester of pregnancy. The median time between onset of malaria before pregnancy and the beginning of pregnancy was 7.6 months with an interquartile range of (4.9–8.5).

**Table 3:** Proportion of pregnant women infected during the first trimester of pregnancy, Sô-Ava and Akassato, Benin 2015–2016

Malaria infection	Pre-pregnancy visit		Visit 1		Visit 2		Visit 3	
	*n*	% (95% CI)	*n*	% (95% CI)	*n*	% (95% CI)	*n*	% (95% CI)
Total	227		234		199		180	
Yes	10	4.41 (2.37; 8.03)	14	5.98 (3.56; 9.88)	10	5.02 (2.71; 9.13)	17	9.44 (5.92; 14.73)

A total of 239 pregnant women were followed during 626.63 person-months, with a median follow-up time of 88 days (Interquartile interval: 65–95)

**Table 4:** Factors associated with the time to onset of the first malaria infection during the first trimester of pregnancy (multivariate Cox model, *N* = 190 pregnant women); Sô-Ava and Akassato, Benin 2015–2016

Variables (terms)		Multivariatble Cox model			
		N	HRa	CI 95%	pvalue
Use of LLIN by the woman the week before the visit		190			
	No	36	1		
	Yes	154	0.38	(0.18–0.80)	< 0.001
Physical integrity of the pregnant woman’s LLIN		190			
	Bad	78	1		
	Good	112	0.59	(0.29–1.19)	0.07
Quantitative bio-efficacy^a^ of the pregnant woman’s LLIN		190	0.97	(0.94–1.00)	0.04
Malaria before pregnancy		190			
	Yes	9	1		
	No	181	0.27	(0.09–0.74)	0.01

*N* Total, *HRa* Hazard ratio adjusted, *CI 95%* Confidence Interval at 95%, *LLIN* Long Lasting Impregnated bedNets, *pvalue* one-sided pvalue

^a^Quantitative bio-efficacy is defined as the proportion of female anopheles who died 24 h after exposure to the LLIN

**Fig. 2:**
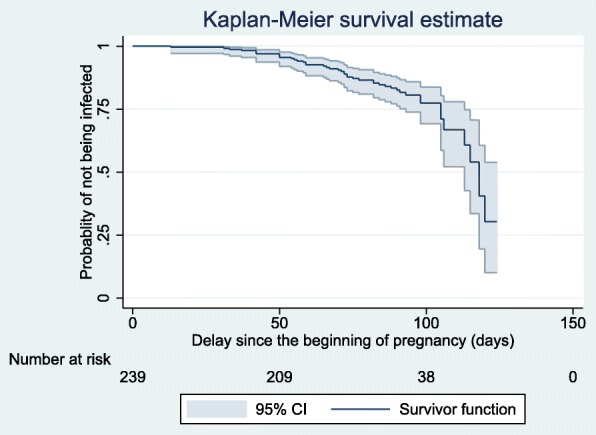
Probability of non-occurrence of malaria infection in women during the first trimester of pregnancy, Sô-ava and Akassato, Benin, 2015–2016

Figure 2 shows the probability of non-occurrence of malaria infection in pregnant women as a function of time during the first trimester of pregnancy. The incidence rate of malaria infections was 7.18 cases per 100 person-months (95% CI: 5.36–9.62).

The group of women not included in the multivariate analysis did not differ significantly from the included pregnant women, according to the 4 variables: age, residence area, gestational rank and malaria before pregnancy (respective *p*-values = 0.48; 0.29; 0.24 and 0.58 respectively). Based on those results, excluding the 50 women from the multivariable model does not seem to have led to a major selection bias.

The variables selected in the final multivariate model respected the proportional hazard asumption assumption according to the Schöenfeld residuals method.

Additional file 1: Table S1 and Table 4 respectively summarize the univariate Cox regression model and the final multivariate Cox model. After adjustment, the use of LLIN by pregnant women was marginally significant, whereas the physical integrity, the LLIN’s quantitative bio-efficacy and malaria infection in pre-pregnancy were significantly associated with the time to onset of the first malaria infection in the first trimester of pregnancy.

We also performed a Poisson model to study the effect of the three indicators of LLIN on the number of malaria infections occurring during the first trimester of pregnancy adjusted for the same covariates. In this analysis (Additional file 2: Table S2), the three indicators of the LLINs (the use, the physical integrity and the quantitative bio-efficacy) were significantly associated with the incidence of malaria infection during the first trimester of pregnancy, (IRRa = 0.40; (0.20–0.78); *p* < 0.003; IRRa = 0.44; (0.24–0.81); *p* < 0.004; IRRa = 0.98; (0.95–0.99); *p* < 0.02 respectively), confirming the results of the Cox model.

## Discussion

The peculiarity of our study was the ability to have followed the women from the pre-conceptional period until the end of the first trimester of pregnancy. This study identified two sets of essential results, i) the refined characterization of the protection given to the pregnant women by the LLIN against malaria infection during the first trimester of pregnancy and ii) new important elements in the reflection on the different indicators of LLIN’s efficiency that characterize properly the real protection of pregnant women against malaria during this critical period.

We observed a general high possession and good use of LLIN by the study pregnant women. This is probably an indication of the success of the LLIN mass distribution campaigns carried out previously in Benin in 2011 and 2014. In all, the Cox model confirmed our working hypothesis that the different indicators were significantly related to the delay in malaria infection. Precisely, good use, good physical integrity and the quantitative bio-efficacy of the LLIN were found to be associated with a later occurrence of the first malarial infection during the first trimester of pregnancy. In this model the physical integrity of the LLIN was associated only marginally significantly with the delay of the first malaria infection but showed significant associations in the Poisson regression model. Five pregnant women had malaria infection twice in our study. Two studies did not find any association between LLIN usage by pregnant women and the risk of malaria infection [21, 22]. Since the effectiveness of LLIN to reduce the malaria burden has been extensively established [23] including in pregnant women [15, 24–28], those result show probably the limit of the two classical indicators of ownership and reported LLIN use the previous night to characterize adequately the optimal protection of the pregnant woman against the occurrence of a malaria infection. All these elements point out the need to consider, in addition to the classical ownership and reported use indicators, the physical integrity and bio-efficacy indicators should be taken into account in evaluation studies of LLIN efficiency to reduce the malaria burden in pregnant women.

In our study, the high proportions of good use and LLINs with good physical integrity are in favor of a good physical barrier conferred by LLIN to the pregnant women during the first trimester of pregnancy. Nevertheless, a more worrying result was the small minority of LLIN reaching the bio-efficacy threshold set by the WHO recommendations. This raises the highly important question of the duration of the LLINs bio-efficacy in field conditions. Given that optimal protection by the LLIN is provided by the combination of its physical barrier and chemical efficacy, we can conclude that the extreme majority of women in the study were not optimally protected against malaria in the first trimester of their pregnancy by the LLIN they used. This important conclusion would have been missed if only based on the ownership and usage classical indicators.

A strength of our study is that for the first time in a malaria cohort, the women were seen at a pre-conceptional stage which allowed to follow them on the very beginning of their pregnancy. This is an important strenght compared to non preconcepional studies. Although our results indicate minimal selection bias, the moderate size of our sample and the particular facies of the study area (lake zone) impose some caution on the representativeness of the results.

## Conclusion

Our study demonstrated for the first time an overall good physical protection against the malaria vector conferred by LLIN in pregnant women in their first trimester of pregnancy, but low chemical protection in our study area. An important conclusion is that, in addition to the traditional indicator of possession of LLINs by pregnant women, it is essential to take into account other indicators such as actual use, physical integrity and bio-efficiency of LLIN, since all these indicators reflect independently from each other the real protection against malaria of this population. Further studies are then needed to assess the generalizability of our results and also to control the duration of the chemical effectiveness of the LLIN distributed on the territory.

## Additional files

Additional file 1: **Table S1.** Factors associated with delay in the first malaria infection during the first trimester of pregnancy, univariate Cox analysis (*N* = 240); Sô-ava and Akassato, Benin 2015–2016. (DOCX 23 kb)

Additional file 2: **Table S2.** Factors associated with the number of malaria infections during the first trimester of pregnancy (multivariate Poisson regression model, *N* = 194 pregnant women); Sô-ava and Akassato, Benin 2015–2016. (DOCX 16 kb)

## Abbreviations

ACT: Artemisinin Combined Therapy
CER-ISBA: Ethics Committee of the Institute of Applied Biomedical Sciences
HRa: Hazard Ratio adjusted
IPTp: Intermittent Preventive Treatment in Pregnant women
IRRa: Incidence Rate Ratio adjusted
LBW: Low Birth Weight
LLIN: Long Lasting Insecticidal Nets
RDT: Rapid Diagnostic Tests
SP: Sulfadoxine - Pyrimethamine
TBS: Thick Blood Smear
WHO: World Health Organization
