# Supplementary material for: Impact of the use and efficacy of long lasting insecticidal net on malaria infection during the first trimester of pregnancy - a pre-conceptional cohort study in southern Benin
Source: BMC Public Health. 2018 Jun 1;18:683. doi: 10.1186/s12889-018-5595-2 (PMC5984809; doi:10.1186/s12889-018-5595-2)
Supplement: Supplementary file 1 — Table S1. Factors associated with delay in the first malaria infection during the first trimester of pregnancy, univariate Cox analysis (N = 240); Sô-ava and Akassato, Benin 2015–2016. (DOCX 23 kb) [file 12889_2018_5595_MOESM1_ESM.docx]

Additional file 1: Table S1: Factors associated with delay in the first malaria infection during the first trimester of pregnancy, univariate Cox analysis (N = 240); Sô-ava and Akassato, Benin 2015-2016

| **Variables (terms)** | | **Malaria infection** | | |
| --- | --- | --- | --- | --- |
|  |  | **HRa** | **IC95 %** | **pvalue** |
|  | |  |  |  |
| **Use of the household’s LLINs the week before the visit** | |  |  | 0.16 |
|  | *No* | 1 |  |  |
|  | *Yes* | 0.69 | (0.33-1.45) |  |
|  |  |  |  |  |
| **Use of the LLIN by the pregnant woman the week before the visit** | |  |  | 0.01 |
|  | *No* | 1 |  |  |
|  | *Yes* | 0.44 | (0.22-0.89) |  |
|  |  |  |  |  |
| **Number of the LLIN per capita (WHO)** | |  |  | 0.30 |
|  | *<0,5* | 1 |  |  |
|  | *>=0,5* | 1.22 | (0.58-2.59) |  |
|  |  |  |  |  |
| **Age in classes** |  |  |  | 0.01 |
|  | *<23 years old* | 1 |  |  |
|  | *(24-27) years old* | 0.98 | (0.50-1.94) |  |
|  | *(28-30) years old* | 0.36 | (0.13-0.97) |  |
|  | *> 30 years old* | 0.32 | (0.09-1.09) |  |
|  |  |  |  |  |
| **Gestational rank** |  |  |  | 0.12 |
|  | *<3 pregnancies* | 1 |  |  |
|  | *>=3 pregnancies* | 0.78 | (0.41-1.51) |  |
|  |  |  |  |  |
| **Marital status** |  |  |  | 0.29 |
|  | *Not married* | 1 |  |  |
|  | *Married* | 1.68 | (0.23-12.30) |  |
|  |  |  |  |  |
| **Ethnic group** |  |  |  | 0.21 |
|  | *Others* | 1 |  |  |
|  | *Toffin* | 1.33 | (0.65-2.72) |  |
|  |  |  |  |  |
| **Education level** |  |  |  | 0.19 |
|  | *Illiterate* | 1 |  |  |
|  | *Literate* | 0.74 | (0.38-1.45) |  |
|  |  |  |  |  |
| **Professional status** | |  |  | 0.45 |
|  | *Unemployed* | 1 |  |  |
|  | *Employed* | 1.07 | (0.33-3.49) |  |
|  |  |  |  |  |
| **Residence area** |  |  |  | 0.22 |
|  | *Land area* | 1 |  |  |
|  | *Lake area* | 1.31 | (0.64-2.70) |  |
|  |  |  |  |  |
| **LLIN received in prenatal consultation** | |  |  | 0.18 |
|  | *No* | 1 |  |  |
|  | *Yes* | 0.71 | (0.38-1.30) |  |
|  |  |  |  |  |
| **Physical integrity of pregnant woman’s LLIN** | |  |  | 0.04 |
|  | *Good* | 1 |  |  |
|  | *Bad* | 1.59 | (0.86-2.93) |  |
|  |  |  |  |  |
| **Physical integrity of household’s LLINs** | |  |  | 0.09 |
|  | *Good* | 1 |  |  |
|  | *Bad* | 0.63 | (0.32-1.24) |  |
|  |  |  |  |  |
| **Quantitative bio-efficacy**^1^ **of the pregnant woman’s LLIN** | | 0.98 | (0.96-1.01) | 0.06 |
|  | |  |  |  |
| **Quantitative bio-efficacy of the household’s LLIN** | | 1.00 | (0.98-1.02) | 0.27 |
|  | |  |  |  |
| **Bio-efficacy of woman’s LLIN according to WHO** | | |  | 0.36 |
|  | *No* | 1 |  |  |
|  | *Yes* | 1.25 | (0.38-4.13) |  |
|  |  |  |  |  |
| **Bio-efficacy of household’s LLINs according to WHO** | |  |  | 0.07 |
|  | *No* | 1 |  |  |
|  | *Yes* | 2.81 | (0.83-9.48) |  |
|  | |  |  |  |
| **Malaria before pregnancy** | |  |  | <0.001 |
|  | *Yes* | 1 |  |  |
|  | *No* | 0.21 | (0.08-0.53) |  |

^1^ Quantitative bio-efficacy is defined as the proportion of female anopheles who died 24 hours after exposure to the LLIN
