# Supplementary material for: Impact of the use and efficacy of long lasting insecticidal net on malaria infection during the first trimester of pregnancy - a pre-conceptional cohort study in southern Benin
Source: BMC Public Health. 2018 Jun 1;18:683. doi: 10.1186/s12889-018-5595-2 (PMC5984809; doi:10.1186/s12889-018-5595-2)
Supplement: Supplementary file 2 — Table S2. Factors associated with the number of malaria infections during the first trimester of pregnancy (multivariate Poisson regression model, N = 194 pregnant women); Sô-ava and Akassato, Benin 2015–2016. (DOCX 16 kb) [file 12889_2018_5595_MOESM2_ESM.docx]

Additional file 2: Table S2: Factors associated with the number of malaria infections during the first trimester of pregnancy (multivariate Poisson regression model, N = 194 pregnant women); Sô-ava and Akassato, Benin 2015-2016

| **Variables (terms)** | | **Final multivariable model** | | | |
| --- | --- | --- | --- | --- | --- |
|  |  | N | IRRa | IC95 % | pvalue |
|  |  |  |  |  |  |
| **Use of LLIN by the woman the week before the visit** |  |  |  |  | 0.003 |
|  | *No* | 35 | 1 |  |  |
|  | *Yes* | 159 | 0.40 | (0.20-0.78) |  |
|  |  |  |  |  |  |
| **Physical integrity of the pregnant woman’s LLIN** |  |  |  |  | 0.004 |
|  | *Bad* | 82 | 1 |  |  |
|  | *Good* | 112 | 0.44 | (0.24-0.81) |  |
|  |  |  |  |  |  |
| **Quantitative bio-efficacy** ^1^**of the pregnant woman’s LLIN** |  | 194 | 0.98 | (0.95-0.99) | 0.02 |

**N**: Total; **IRRa**: Incidence Rate Ratio adjusted; **CI 95 %: Confidence Interval at 95 %; LLIN**: Long Lasting Impregnated bedNets; **pvalue:** one-sided pvalue

^1^ Quantitative bio-efficacy is defined as the proportion of female anopheles who died 24 hours after exposure to the LLIN
